# Ameloblastic Fibroma: Clinical Perspectives on a Rare Odontogenic Lesion

**DOI:** 10.7759/cureus.88629

**Published:** 2025-07-23

**Authors:** Veerendranath Reddy Panthula, G V Reddy, M R Haranadha Reddy, Sarah Fatima, Suhail Mir, Shivani G, Vyshnavi Shingade

**Affiliations:** 1 Department of Periodontics, Panineeya Mahavidyalaya Institute of Dental Sciences & Research Centre, Hyderabad, IND; 2 Department of Oral and Maxillofacial Surgery, Panineeya Mahavidyalaya Institute of Dental Sciences & Research Centre, Hyderabad, IND

**Keywords:** ameloblastic fibroma, enucleation, histopathology, odontogenic tumor, supernumerary tooth

## Abstract

Ameloblastic fibroma (AF) is a rare non-cancerous growth in the jaw that involves abnormal development of the epithelial and connective tissue parts of a tooth. A 33-year-old female presented with bleeding gums, mobile anterior mandibular teeth, and persistent halitosis. Initial oral examination and orthopantomogram revealed a unilocular radiolucent lesion with partially sclerotic borders, associated with an unerupted supernumerary tooth in the mandibular ramus. Computed tomography demonstrated cortical plate thinning around the lesion. Given the clinical and radiographic presentation, a differential diagnosis included odontogenic keratocyst and ameloblastoma. Histopathological evaluation following incisional biopsy and surgical enucleation performed under local anesthesia confirmed the diagnosis of AF, characterized by distinctive epithelial islands and mesenchymal stroma. This case highlights the imperative for meticulous differential diagnosis, supported by histopathological examination, to distinguish AF from related lesions with overlapping features. Given its potential for recurrence and the rare risk of malignant transformation into ameloblastic fibrosarcoma, long-term radiologic surveillance remains essential for optimal patient management.

## Introduction

Ameloblastic fibroma (AF) is a rare benign odontogenic neoplasm categorized as a mixed tumor owing to the concurrent proliferation of odontogenic epithelium and mesenchymal tissue, without evidence of dental hard tissue formation. This defining characteristic distinguishes AF from other odontogenic lesions, such as ameloblastic fibro-odontoma and odontoma, which are characterized by the development of enamel and dentin matrices [1].

First described in the early 20th century, the biological behavior of AF is notably less aggressive than that of ameloblastoma, making its identification essential for appropriate therapeutic planning [2]. AF is part of a group of mixed odontogenic tumors, which includes ameloblastic fibrodentinoma, ameloblastic fibro-odontoma, and odontomas [3]. Some researchers believe these tumors may represent different stages in the development of a single disease process.

According to Nalabolu et al. [4], odontogenic tumors account for approximately 2.17% of oral biopsies, with AF comprising only 0.6% of this group. While AF typically appears in the posterior mandible of younger patients, variations in age and clinical presentation, as observed in the present case, underscore the importance of carefully evaluating distinguishing features during diagnosis.

## Case presentation

A 33-year-old female reported to the Department of Periodontics and Implantology, Panineeya Mahavidyalaya Institute of Dental Sciences and Research Centre, with a chief complaint of bleeding gums, tooth mobility in the anterior mandibular region, and chronic halitosis. The patient was otherwise healthy, with no relevant medical, surgical, or medication history.

Clinical examination

Extraoral evaluation revealed symmetrical facial features with no lymphadenopathy or paresthesia. Intraoral inspection showed erythematous gingiva and grade I mobility in teeth 32, 31, 41, and 42. A complete oral prophylaxis was performed, and the patient was advised to undergo an orthopantomogram (OPG).

Radiographic evaluation

**Figure 1:**
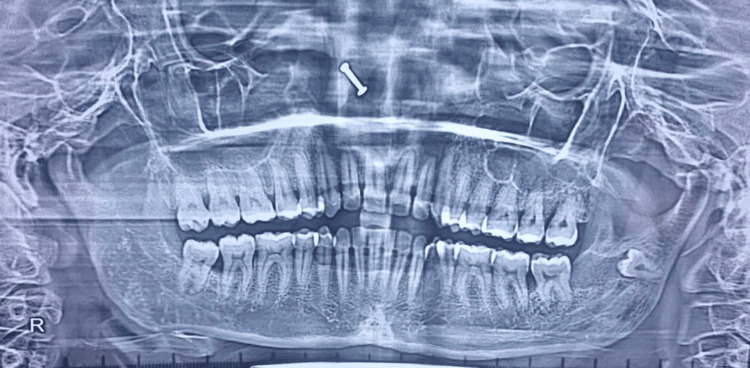
Dental panoramic radiograph.

The OPG revealed a well-defined unilocular radiolucency in the left mandibular ramus, extending from the distal surface of tooth 38 to the inferior border of the mandible (Figure 1).

**Figure 2:**
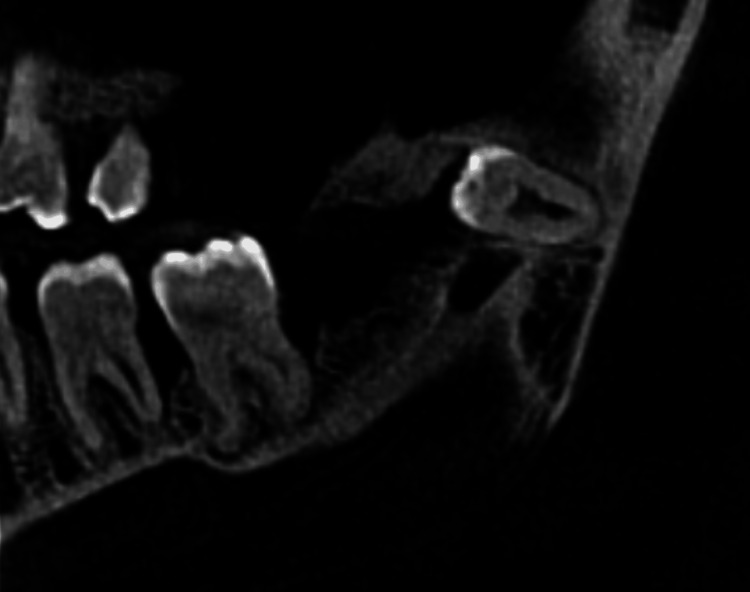
No root resorption was noted.

The lesion was demarcated by sclerotic borders and encompassed an irregular radiolucent mass containing an unerupted supernumerary tooth near the base of the ramus. Notably, no root resorption was observed (Figure 2).

**Figure 3:**
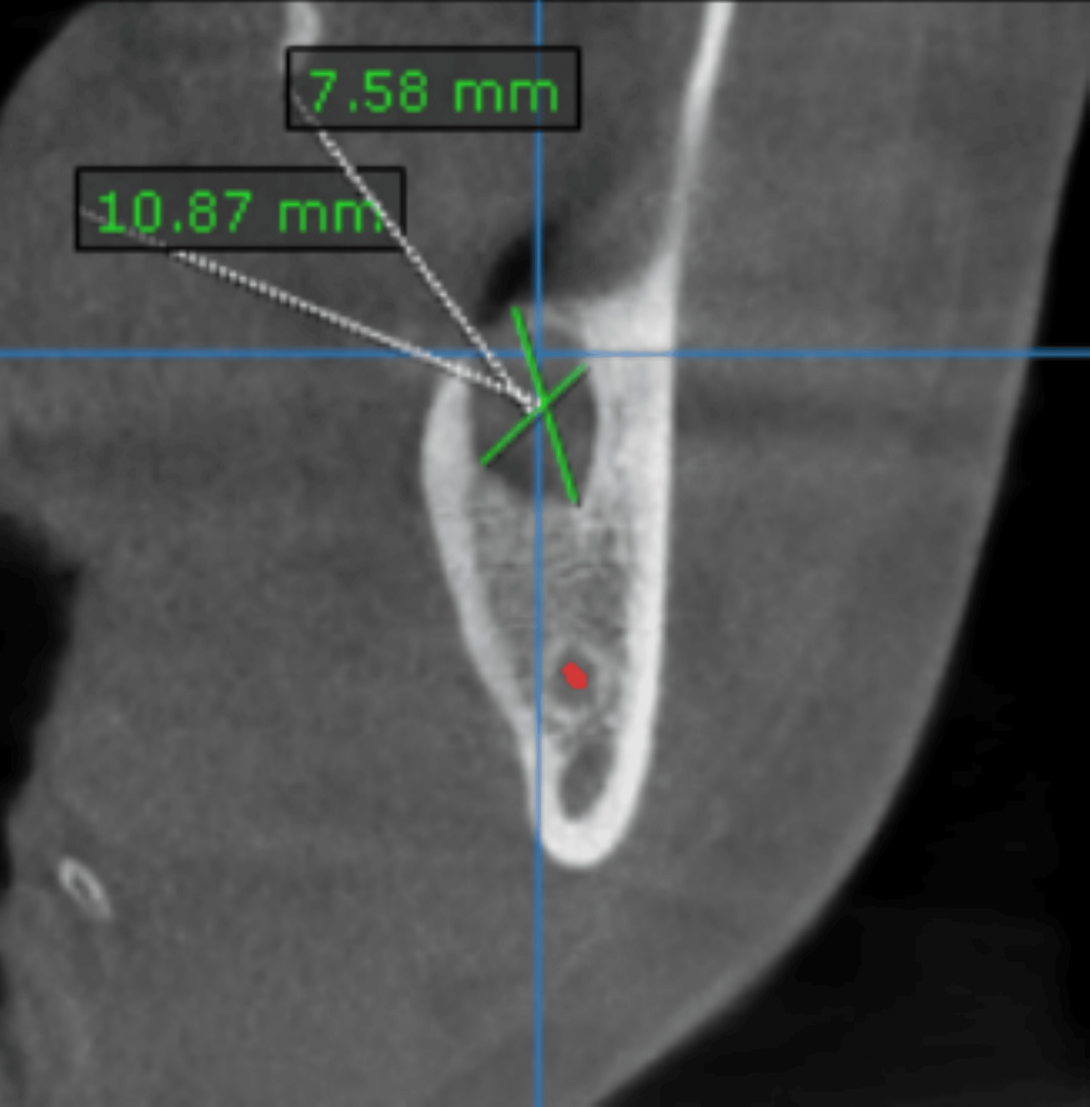
A cross-sectional view showing the dimensions of the lesion, along with the perforation of the lingual cortical plate.

**Figure 4:**
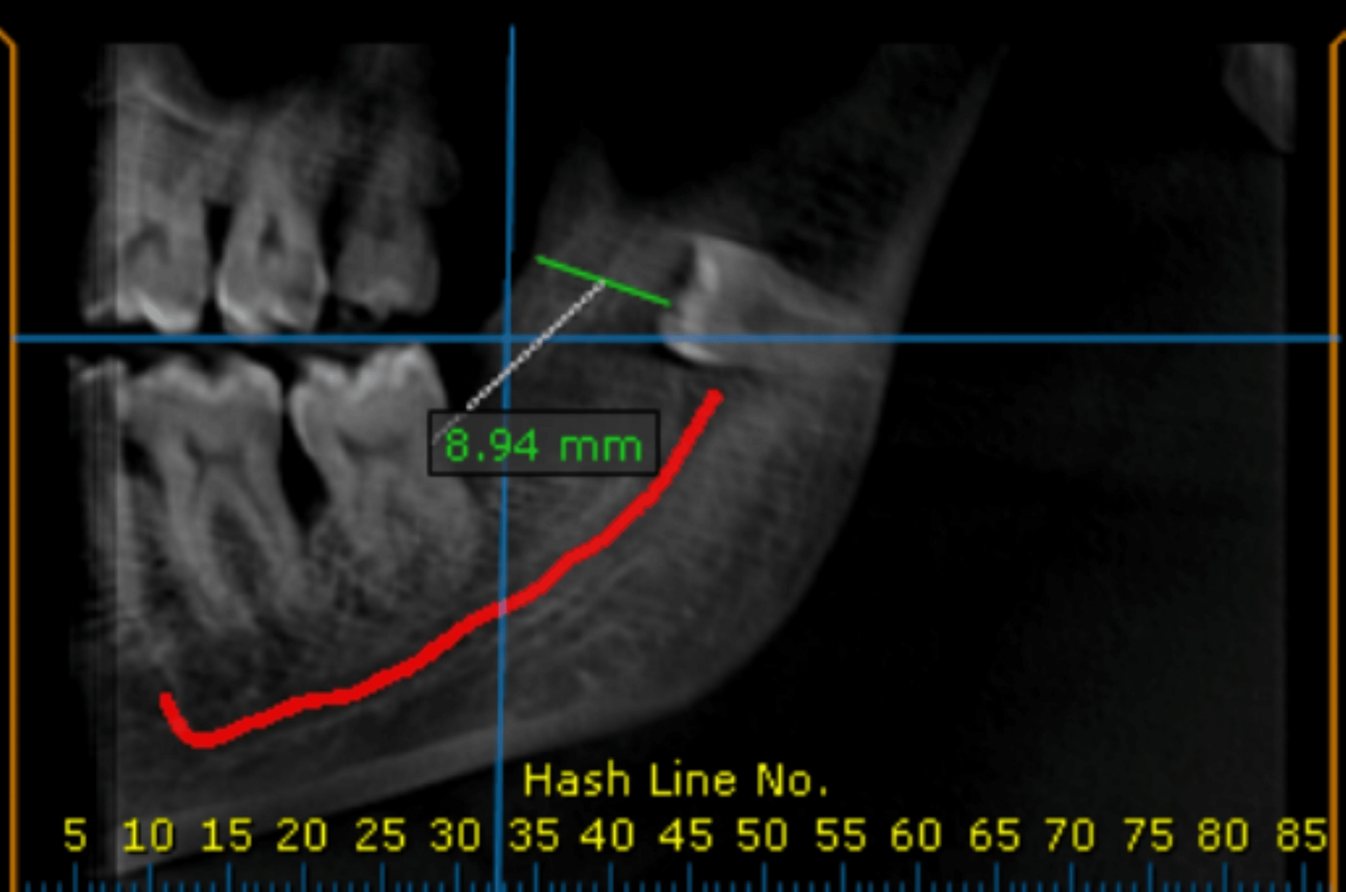
A panoramic view showing the distance of the supernumerary unerupted tooth from the anterior border of the ramus.

Subsequent cone beam computed tomography (CBCT) showed a hypodense lesion with internal iso density, thinning of the lingual cortical plate, and proximity of the inferior alveolar nerve. A cross-sectional view indicated dimensions of 10.87 × 7.58 mm (Figure 3), with lingual cortical plate perforation. The lesion measured approximately 14.18 mm anteroposteriorly, and the supernumerary tooth lay 8.94 mm from the anterior border of the mandibular ramus (Figure 4).

Surgical procedure

**Figure 5:**
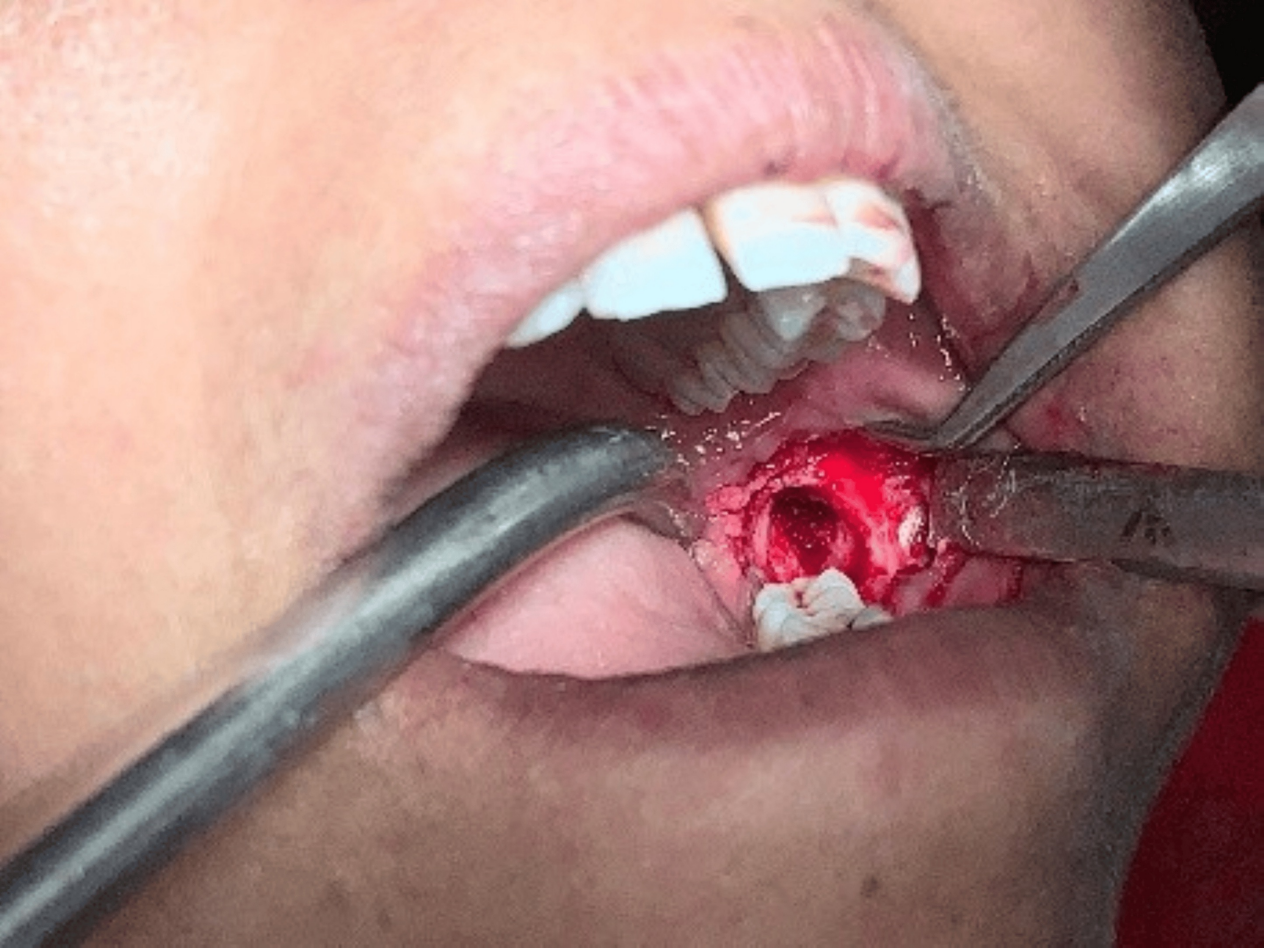
Intra-operative picture of the patient.

Following informed consent and preoperative evaluation, an excisional biopsy was performed under local anesthesia. Tooth 38 was extracted to access the lesion, and a small bony window was created distal to tooth 38 using a round bur. The solid tumor was fully enucleated, followed by thorough curettage. The supernumerary tooth was preserved to prevent mandibular fracture, given its proximity to the inferior border and existing cortical perforation (Figure 5).

Investigations

Two specimens measuring approximately 1.5 × 3 mm and 1.8 × 3 mm were sent for histopathological analysis. A gel foam impregnated with 5-fluorouracil was placed in the defect, and primary closure was achieved with 3-0 black braided silk sutures. Postoperative care included a course of antibiotics and analgesics.

Histopathology

Microscopic Findings

**Figure 6:**
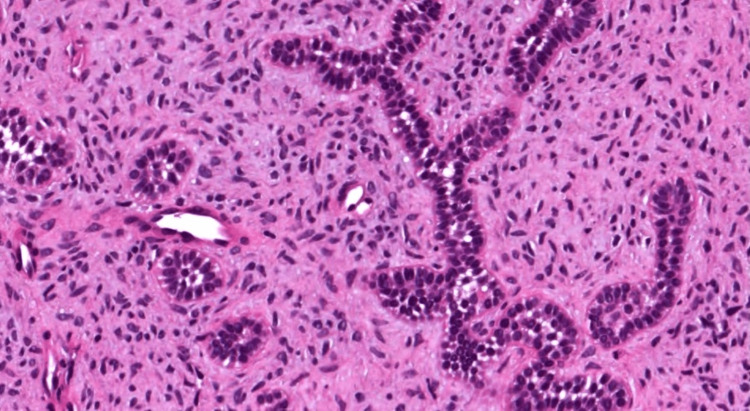
Histopathological picture showing features of ameloblastic fibroma.

Histological examination revealed features characteristic of ameloblastic fibroma. The connective tissue exhibited a cell-rich ectomesenchymal stroma that resembled dental papilla, interspersed with numerous strands, cords, and islands of odontogenic epithelium. These epithelial structures were predominantly two cells thick and composed of cuboidal to columnar cells, often arranged in anastomosing patterns (Figure 6).

The absence of enamel or dentin formation further confirmed the diagnosis, distinguishing it from other mixed odontogenic tumors such as ameloblastic fibro-odontoma or odontoma.

The epithelial and mesenchymal components appeared well-differentiated, with no signs of cytological atypia or malignancy. No calcifications, ghost cell changes, or dysplastic dentin were noted in this case.

Final diagnosis

Based on the histological architecture and clinical correlation, a diagnosis of ameloblastic fibroma was established.

Follow-up

One week postoperatively, the patient reported mild swelling and reduced mouth opening. Sutures were removed, and physiotherapy with mouth-opening exercises was initiated. At the 15-day follow-up, the swelling had resolved, and the patient regained full mandibular mobility (~50 mm interincisal opening). Radiographic follow-ups, including OPG and CBCT, at one, three, and six months showed progressive bone regeneration. The patient was advised to continue annual follow-ups for five years to monitor for recurrence.

## Discussion

This case underscores a rare clinical presentation of AF in a 33-year-old female, deviating from the typical age distribution, which predominantly favors younger patients. The majority of AF cases, nearly 72.4%, are reported in the first and second decades of life, with a mean age of approximately 14.8 years [2-4]. This case is unusual because the tumor appeared later than expected and was associated with an extra tooth.

Radiographically, the lesion exhibited a well-defined unilocular radiolucency with sclerotic margins, features typically seen in early or smaller AFs. As documented by Chen et al. [5], smaller lesions (<4 cm) tend to be unilocular, while larger ones may become multilocular. The presence of an unerupted supernumerary tooth adds complexity and mimics presentations seen in dentigerous cysts or ameloblastic fibro-odontomas. The lingual cortical plate perforation and proximity to the inferior alveolar nerve further highlight the lesion’s expansile but non-invasive behavior, characteristic of benign odontogenic neoplasms.

Histopathology remains the gold standard in distinguishing AF from its odontogenic mimickers. In this case, the tumor demonstrated the classic features of AF: branching cords and islands of odontogenic epithelium within a primitive ectomesenchymal stroma that resembled dental papilla. Importantly, the absence of mineralized tissue, ghost cells, or enamel/dentin formation excluded diagnoses such as ameloblastic fibro-odontoma or calcifying odontogenic cyst [3]. The lesion also lacked cytologic atypia, helping to rule out ameloblastic fibrosarcoma.

Differential diagnosis initially included odontogenic keratocyst and ameloblastoma, based on radiographic appearance [6]. However, the unilocular pattern, histologic findings, and absence of aggressive clinical features favored AF. These distinctions are critical, as treatment approaches vary significantly between benign neoplasms and cystic or malignant counterparts [7-9].

In terms of management, a conservative surgical approach was adopted, involving complete enucleation without removal of the supernumerary tooth, to preserve mandibular integrity. This decision aligns with literature recommendations for benign AFs in younger or otherwise healthy patients, particularly where anatomical risk is high [5-7]. Radical surgery is generally reserved for recurrent, large, or histologically ambiguous cases.

While AF is benign, recurrence has been reported in up to 33% of cases, and malignant transformation into ameloblastic fibrosarcoma, though rare, is a recognized concern [10,11]. Accordingly, the patient was enrolled in a systematic follow-up regimen, incorporating radiologic evaluations scheduled at progressively extended intervals over a five-year period, in alignment with established clinical guidelines.

This case reinforces the clinical value of integrating imaging, histology, and surgical judgment in managing odontogenic tumors. It contributes to the growing literature advocating individualized, tissue-preserving management of AF, especially in older patients with anatomically complex lesions.

## Conclusions

AF, though rare, should remain an important differential consideration in the evaluation of radiolucent jaw lesions, particularly when associated with unerupted teeth. This case underscores the diagnostic complexity of AF, especially in atypical age groups, and the essential role of histopathology in achieving a definitive diagnosis. Conservative surgical management, guided by precise radiographic and clinical assessment, offers favorable outcomes, provided vigilant long-term follow-up is maintained. Continued awareness and documentation of such unusual presentations enhance our understanding of this enigmatic odontogenic tumor and guide future treatment protocols.
